# A stacked ensemble machine learning model for the prediction of pentavalent 3 vaccination dropout in East Africa

**DOI:** 10.3389/fdata.2025.1522578

**Published:** 2025-04-07

**Authors:** Meron Asmamaw Alemayehu, Shimels Derso Kebede, Agmasie Damtew Walle, Daniel Niguse Mamo, Ermias Bekele Enyew, Jibril Bashir Adem

**Affiliations:** ^1^Department of Epidemiology and Biostatistics, College of Medicine and Health Sciences, Institute of Public Health, University of Gondar, Gondar, Ethiopia; ^2^Department of Health Informatics, School of Public Health, College of Medicine and Health Science, Wollo University, Dessie, Ethiopia; ^3^Department of Health Informatics, School of Public Health, Asrat Woldeyes Health Science Campus, Debre Berhan University, Debre Birhan, Ethiopia; ^4^Department of Health Informatics, College of Medicine and Health Sciences, Arba Minch University, Arba Minch, Ethiopia; ^5^Department of Public Health, College of Medicine and Health Science, Arsi University, Asella, Ethiopia

**Keywords:** Penta 3 vaccination, stacked ensemble model, SHAP values, East Africa, machine learning

## Abstract

**Introduction:**

Vaccination is critical for reducing childhood mortality, yet completion rates for the third dose of the pentavalent vaccine (Penta 3) in East Africa remain inadequate. This study aims to predict Penta 3 vaccination dropout using a stacking ensemble machine learning model with Demographic and Health Survey (DHS) data. The objective is to identify predictors of dropout and enhance intervention strategies.

**Methods:**

The study utilized seven base machine learning algorithms to create a stacked ensemble model with three meta-learners: Random Forest (RF), Generalized Linear Model (GLM), and Extreme Gradient Boosting (XGBoost). The H2O package facilitated the development of base learners and the stacking of super learners. Feature selection (FS) and comparisons were performed using the LASSO and Boruta algorithms. The selected features were one-hot encoded, and ordinal encoding was applied where appropriate. Hyperparameter optimization (HPO) and comparisons were conducted using grid search and random search. Model performance was assessed using five key metrics, including accuracy and the area under the curve (AUC). SHAP (Shapley Additive Explanations) values were used to interpret the model outputs and identify influential predictors. The experimental design was employed to present the results.

**Results:**

Four experiments were conducted to evaluate feature selection and HPO methods. All stacked ensemble models outperformed individual learners, with the XGBoost meta-learner optimized with grid search and LASSO FS achieving the highest performance: 93.9% accuracy and 99.4% AUC. While RF and GLM meta-learners were also evaluated, they were outperformed by the XGBoost meta-learner. SHAP analysis revealed key features influencing Penta 3 dropout, including the place of delivery, decision-making autonomy, the mother's level of earning, and healthcare access. Home delivery increased the risk of dropout, while postnatal care by midwives and health insurance coverage lowered dropout likelihood.

**Conclusion and recommendation:**

This study provides insights into the factors influencing Penta 3 vaccination dropout in East Africa. To reduce dropout rates, interventions should focus on enhancing maternal livelihood opportunities, improving healthcare access in rural areas, and promoting institutional deliveries.

## Introduction

Childhood vaccination dropout is an ongoing public health challenge, particularly in sub-Saharan Africa, where achieving high vaccination coverage remains a priority. Among the vaccines included in the Expanded Program on Immunization (EPI), the pentavalent vaccine is essential for protecting children from five life-threatening diseases: diphtheria, tetanus, whooping cough, hepatitis B, and Haemophilus influenzae type B. While significant progress has been made in expanding vaccine coverage across many regions, the third dose of the pentavalent vaccine (Penta3) continues to see relatively low completion rates, especially in low- and middle-income countries, including those in East Africa. The failure to complete the full pentavalent series, particularly the third dose, poses serious public health risks, as incomplete vaccination leaves children vulnerable to preventable diseases and undermines efforts to achieve herd immunity. Addressing this dropout issue is crucial for improving vaccination coverage and preventing further strain on healthcare systems in the region (World Health Organization, 2023).

Various factors contribute to the high rates of pentavalent vaccine dropout. Logistical challenges, including limited access to healthcare facilities, transportation barriers, and inadequate infrastructure in rural areas, make it difficult for parents to return for subsequent doses. Additionally, misinformation and vaccine hesitancy, often fueled by myths and misinformation circulating through the community and social media, lead many caregivers to question the safety and necessity of vaccines. These issues are exacerbated by weak health systems that struggle with maintaining consistent vaccine supplies and outreach programs. Consequently, children in the region remain at heightened risk for vaccine-preventable diseases, and healthcare systems are further burdened by the failure to complete vaccination schedules (Tsegaw et al., 2024; World Health Organization, 2023).

This study aims to develop and evaluate a stacked ensemble machine learning model that can accurately predict pentavalent 3 vaccination dropout in East Africa and address these challenges by developing a robust prediction model. The model's goal is to identify and analyze the key predictors contributing to vaccination dropout, with the intention of providing actionable insights for targeted public health interventions. By employing a data-driven approach, this study fills a significant gap in the existing literature on vaccine dropout, offering a more objective and precise understanding of the issue. Additionally, it showcases the utility of machine learning models in public health research, demonstrating how such tools can be applied to complex, real-world problems that are difficult to address using traditional methods.

This research makes several important contributions. First, it introduces the use of a stacked ensemble machine learning model to predict Penta3 dropout, a novel methodology in the context of vaccine dropout prediction. This model integrates multiple machine learning algorithms to improve prediction accuracy and robustness, offering a significant advancement over stand-alone models, which have been more commonly used in similar studies. Second, this study has practical implications for policymakers and public health organizations, including the World Health Organization (WHO), that are working to improve vaccination completion rates. Despite the WHO's goal of achieving 90% coverage for the third dose of the pentavalent vaccine, many East African countries are still far from this target (World Health Organization, 2020, 2024). By identifying the socioeconomic, geographic, and healthcare system factors that influence vaccine dropout, this study provides evidence-based recommendations for interventions that are tailored to the specific needs and challenges of the region.

From a theoretical perspective, this research contributes to the growing field of machine learning applications in public health. By using a stacked ensemble framework, the study demonstrates how this technique can capture complex, non-linear relationships between various factors influencing vaccination behaviors. The approach used in this study is not only applicable to Penta3 dropout but also has the potential to inform similar studies in other areas of public health where predicting health-related behaviors can enhance intervention strategies. Additionally, this work offers new insights into the theoretical understanding of vaccination behavior by analyzing how different factors, ranging from socio-economic to health system-related, interact to affect vaccination completion rates.

In conclusion, this study presents a novel machine learning-based framework for predicting Penta3 dropout in East Africa, addressing a gap in existing research while providing a detailed analysis of the factors contributing to vaccination dropout. The insights gained from this study can inform future research and public health policies aimed at improving vaccination coverage and reducing dropout rates. Ultimately, the findings have the potential to guide targeted interventions that can contribute to better child health outcomes in East Africa and other similar regions around the world.

### Related works

The prediction of vaccination dropout using machine learning (ML) techniques has gained increasing attention in recent years. Various studies have explored different methods for predicting vaccination behavior and outcomes. However, most of these studies focus on overall vaccine dropout (not specific vaccines), use statistical methods, focus on a single country, do not use model interpretability analysis like SHAP, and/or rely on single machine learning algorithms, often limiting their predictive power by not leveraging the strengths of multiple algorithms through ensemble learning.

One early work by Chandir et al. (2018) applied decision tree-based models to predict childhood vaccination dropout in low-resource settings. The model showed moderate success, particularly in identifying key predictors, such as maternal education and distance to health facilities, but struggled with generalization across different datasets. This study demonstrated the potential of ML models but highlighted the need for more sophisticated approaches to handle complex socio-demographic patterns. Similarly, a study by Kayembe-Ntumba et al. (2022) used logistic regression models to predict overall vaccination dropouts, focusing on a few socio-economic factors such as rural residence, unavailability of seats, and lack of a reminder system. While the study succeeded in identifying high-risk groups for dropout, the model lacked flexibility and failed to account for non-linear relationships between variables, reducing its overall predictive performance.

More recent studies have turned to ensemble methods to improve model performance. For instance, Demsash et al. (2023) employed Random Forests (RF) and Gradient Boosting Machines (GBM) to predict childhood vaccination in Ethiopia, demonstrating an increase in accuracy compared to single models. However, their study was limited since only one country was involved (i.e., Ethiopia), it was not focused on a specific childhood vaccine, no model interpretability method was employed, and only individual models were evaluated. Similarly, Nwachukwu (2024) applied machine learning techniques to predict immunization completion in Ogun State, Nigeria. The study employed Logistic Regression, Support Vector Machine (SVM), and K-Nearest Neighbors (KNN) models to analyze immunization patterns using retrospective data from 8,808 immunization records. Logistic Regression was favored with an accuracy of 99.77%, outperforming SVM and KNN. While the study provided valuable insights into immunization in a specific region of Nigeria, it lacked other strong ML models such as XGBoost and RF and also lacked model interpretability tools such as SHAP analysis and did not focus on vaccination dropout explicitly, but rather on overall completion rates. Furthermore, the data was limited to a single locality within Nigeria, limiting its generalizability to broader regions like East Africa.

In a separate study conducted in The Gambia, Ntenda et al. (2022) utilized Generalized Estimating Equation (GEE) models to examine the determinants of pentavalent and measles vaccination dropouts using data from the 2019–20 Gambia Demographic and Health Survey. This study identified key factors influencing dropout rates, such as antenatal care attendance, possession of a health card, and urban residency. While the study was important for highlighting vaccination challenges in The Gambia, it focused primarily on statistical methods and did not incorporate advanced machine learning techniques. Additionally, the scope was limited to The Gambia, without extending to other East African countries.

In contrast to these approaches, our study takes a more advanced approach by employing a stacked ensemble model, integrating predictions from multiple base learners such as Naive Bayes, Random Forests, Gradient Boosting Machines, eXtreme Gradient Boosting, and Deep Learning, among others. The novelty of our approach lies primarily in the stacking process, where a meta-learner is used to combine the predictions of base models, leveraging the strengths of each learner while mitigating their weaknesses. This model architecture allows for enhanced predictive accuracy and robustness, particularly in handling the complex, non-linear relationships often present in socio-demographic data. Previous studies, such as those by Hu et al. (2021), have shown that stacking models outperform traditional ML algorithms in healthcare settings, but their focus was limited to predicting hospital admissions rather than vaccination dropout. By extending this methodology to the prediction of Pentavalent 3 vaccination dropout, we address a critical gap in the literature.

Moreover, by leveraging comprehensive, high-quality, and representative data from the DHS, we enhanced model interpretability and transparency using SHAP analysis, which is not applied in the related works mentioned. Our study's unique focus on Pentavalent 3 dropout across multiple East African countries makes it a significant contribution compared to single-country or general vaccination studies. These combinations not only improve predictive performance but also provide insights into the key drivers of Penta 3 vaccination dropout in East Africa, making it highly relevant for policymakers and public health practitioners working toward vaccine coverage in the region.

## Methods

### Data source

The study utilized data from the Demographic and Health Surveys (DHS) conducted across multiple East African countries. DHS surveys are nationally representative and provide comprehensive information on health indicators, including vaccination coverage and demographic characteristics. Specifically, we accessed the latest available DHS datasets for each country in the region, ensuring consistency and relevance across the study period. These datasets are publicly accessible and rigorously collected using standardized methodologies, which enhances the reliability and comparability of our findings. By leveraging DHS data, we aimed to capture the diverse socio-demographic profiles and health service utilization patterns that influence vaccination behaviors in East Africa. Ethical considerations regarding data use and participant anonymity were adhered to, respecting the confidentiality and rights of survey respondents (The DHS Program, 2024).

### Modeling software and packages

The machine learning analysis for this project was conducted using the R programming language, primarily in a Google Collaboratory environment. The h2o package was utilized to implement a stacked ensemble model consisting of seven base learners.

The integration of these tools provided a robust framework for model development and optimization. The choice of Collaboratory facilitated efficient computation without the need for local hardware resources, ensuring scalability and accessibility in the research workflow.

### Study variables

#### Target

The primary outcome variable examined in this study was Penta 3 vaccination dropout, defined as the failure to complete the third dose of the pentavalent vaccine within the recommended timeframe (1 = dropped out and 0 = received/completed).

#### Features

Predictive variables encompassed a wide array of socio-demographic, economic, and geographic factors known to influence vaccination behaviors. These included maternal age, educational attainment, household income, urban or rural residence, distance to the nearest health facility, and accessibility to healthcare services. Each variable was carefully selected based on its theoretical relevance and empirical evidence linking it to vaccination uptake and completion rates in low-resource settings. The complete list of features and their label is presented in the Supplementary Table 1.

### Data preprocessing

To prepare DHS data for a stacked ensemble machine learning model, comprehensive preprocessing is required. Data cleaning, addresses inconsistencies, duplicates, and incorrect values, ensuring that only high-quality information informs model training. Target and feature engineering refines both target variables and predictors to optimize predictive accuracy. This includes techniques like lumping categories in sparse variables, feature selection with Boruta or Lasso regularization for relevance, feature encoding (one-hot, ordinal, label), and removing highly correlated features to reduce multicollinearity. Handling missing values involves imputing or, if appropriate, removing missing data points to preserve dataset integrity, minimizing biases from incomplete entries (Luengo et al., 2020).

Moreover, splitting the data into training and test sets provides a framework for evaluating model performance, ensuring that the ensemble generalizes well across unseen data. Here, a carefully designed resampling strategy enhances model robustness by creating training subsets that reduce overfitting, which is particularly important in ensemble models that combine predictions from diverse learners. Finally, managing imbalanced data ensures that classes in the target variable are proportionally represented, typically through techniques like the Synthetic Minority Over-sampling Technique (SMOTE) or under-sampling, which prevents dominant classes from skewing model predictions. Together, these preprocessing steps create a balanced, well-prepared dataset, critical for building an effective stacked ensemble model that delivers robust predictions from large datasets like DHS data (Werner de Vargas et al., 2023).

### Base learners and stacked ensemble model

In this study used a stacked ensemble machine learning model that integrates the predictions of seven distinct base learners: Naive Bayes (NB), Generalized Linear Model (GLM), Decision Tree (DT), Deep Learning (DL), Random Forest (RF), Gradient Boosting Machine (GBM), and Extreme Gradient Boosting (XGB). This ensemble approach is designed to enhance predictive performance by leveraging the unique strengths of each individual model while mitigating their weaknesses.

**NB** is a probabilistic classifier based on Bayes' theorem, which assumes independence among features. This model is particularly effective for text classification tasks and performs well with smaller datasets. The **GLM** is a flexible extension of traditional linear regression that accommodates various types of response variables through the use of different link functions. This model facilitates the interpretability of coefficients, enabling insights into the impact of predictors. A **DT** model structures data through a tree-like diagram, making decisions based on the most significant attribute at each node. While easy to interpret and visualize, decision trees can be prone to overfitting if not managed appropriately. **DL** employs neural networks with multiple layers to identify complex patterns in high-dimensional data. While deep learning excels in tasks involving images and text, it typically requires substantial datasets and computational resources. **RF** is an ensemble model that constructs multiple decision trees using random subsets of the training data and features. This method enhances predictive accuracy by reducing overfitting through the aggregation of predictions from individual trees. **GBM** is an ensemble learning method that builds models sequentially, primarily used for regression and classification tasks. It combines multiple weak learners, typically decision trees, to create a strong predictive model by optimizing the model's weights based on the errors of previous iterations. This approach enhances accuracy and reduces prediction errors over time. **XGoost** is a powerful boosting algorithm that builds models sequentially, with each new model addressing the errors made by its predecessor. Renowned for its efficiency and accuracy, XGBoost has demonstrated exceptional performance in various machine learning model comparisons (Shetty and Whitfield, 2023).

### Stacking process

The process of stacking involves several key steps. First, each of the seven base learners are trained on the same training dataset. This initial training phase allows each model to learn from the data, capturing different patterns and relationships. The predictions generated by these models are stored for subsequent use, forming a diverse set of outputs. The next step involves creating a new feature set based on the predictions from the base learners. While some R packages (like h2o) provide a built-in mechanisms to automatically create meta-features as part of their stacking implementations, others (like caret and mlr3) do not. In these cases, predictions must be manually extracted and assembled from base learners into a new feature set for the meta-learner. In this study, the “h2o.stackedEnsemble” function of the “h2o” package automatically generates these predictions from the trained base learners, creating what is often referred to as meta-features. When cross-validation is used during the training of base learners, h2o ensures that the meta-features are based on out-of-fold predictions to prevent data leakage, thereby maintaining the integrity of the validation process.

Next, a meta-learner algorithm, which can be another model such as logistic regression, random forest, or eXtream gradient boosting, is then selected and trained on the meta-features. During this phase, the meta-learner learns how to optimally combine the outputs of the base learners to produce a final prediction. The choice of meta-learner can influence the performance of the ensemble, as it determines how to weigh the contributions from each base model. Lastly, the trained meta-learner produces the final predictions for unseen data. When new input data is provided, the base learners generate their respective predictions, which are then fed into the meta-learner. The meta-learner synthesizes these inputs, producing a robust final prediction that combines the insights gained from all base learners (Verma et al., 2024).

**Figure 1:**
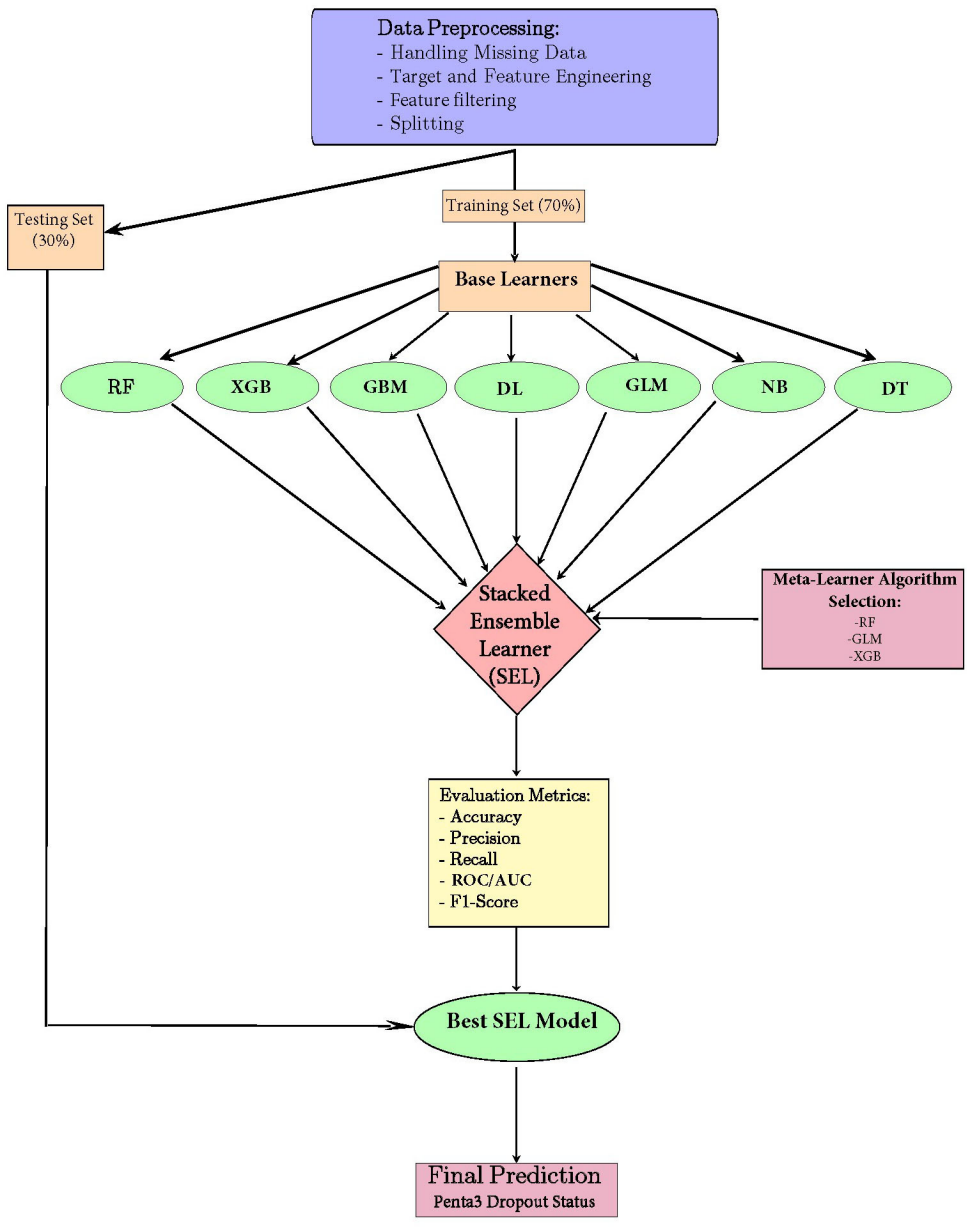
Model-building architecture for a stacked ensemble machine learning model for the prediction of pentavalent 3 vaccination dropout in East Africa.

Stacked ensemble model offers numerous advantages, including improved performance through the integration of diverse model strengths, which enhances predictive accuracy and robustness. By leveraging multiple algorithms, the ensemble approach reduces the risk of overfitting, as it combines the strengths of various models while minimizing their individual weaknesses. Additionally, the flexibility of the stacking framework allows for the inclusion of various types of base learners, enabling researchers to tailor their ensemble to the specific characteristics of their dataset and the nature of their predictive task. Overall, stacked ensembles represent a powerful and versatile strategy for achieving superior performance in machine learning applications (Dey and Mathur, 2023). An architecture representing the model building process is presented in Figure 1.

### Hyperparameter optimization (HPO) and performance metrics

The hyperparameter optimization (HPO) process for a stacked model with seven base learners involves multiple stages, each aimed at optimizing the performance of the model ensemble. First, individual base learners are trained and tuned separately. Each learner has its own set of hyperparameters, such as learning rate, regularization strength, number of estimators, and depth of trees in case of tree-based algorithms.

This study experimented with grid search HPO and systematic random search HPO, both combined with cross-validation, to compare and identify the optimal hyperparameters for each base learner. Cross-validation helps prevent overfitting, ensuring that the selected hyperparameters generalize well to unseen data.

After tuning the base learners, their predictions on the training data set are used as input features for the second layer of the stacked model, typically referred to as the meta-learner. The meta-learner is another machine learning algorithm tasked with combining the predictions from the base learners in a way that minimizes overall prediction error. The hyperparameters of this meta-learner are crucial as well, and they require tuning similar to the base learners. This can be done independently or as part of a larger optimization process that considers both base and meta-learner hyperparameters simultaneously.

During the entire process, cross-validation is employed at different stages to prevent information leakage from the base learners to the meta-learner. This is often done using techniques like k-fold cross-validation or nested cross-validation, where the base learners are trained and tuned in the inner loop, while the meta-learner is trained and tuned in the outer loop. Once the final hyperparameters for both the base learners and the meta-learner are identified, the model is retrained on the full training dataset using the optimized settings. The final stacked model is then evaluated on a held-out test set to ensure robust performance. Hyperparameter tuning for stacked models, while complex, aims to exploit the strengths of each base learner and combine them in a way that maximizes predictive accuracy while minimizing overfitting (Zhang et al., 2023).

To evaluate the performance of the stacked ensemble model, several metrics were employed, including accuracy, precision, recall, area under the receiver operating characteristic curve (AUC-ROC), and F1 score. **Accuracy** measures the proportion of true positive and true negative predictions among the total number of instances examined, providing a general indication of model effectiveness. However, it may not adequately reflect performance in imbalanced datasets, prompting the use of additional metrics.

**Precision**, also known as positive predictive value, quantifies the number of true positive predictions divided by the sum of true positives and false positives. This metric highlights the model's ability to correctly identify positive instances, which is particularly important in contexts where the cost of false positives is high. **Recall**, or sensitivity, measures the proportion of true positive predictions relative to the total number of actual positives, thus providing insight into the model's effectiveness in capturing all relevant cases. The **AUC-ROC** metric assesses the model's ability to distinguish between positive and negative classes across various threshold settings, summarizing its performance in terms of both sensitivity and specificity. Finally, the **F1 score** is the harmonic mean of precision and recall, offering a balance between the two metrics and providing a single measure to evaluate model performance, particularly in scenarios where false negatives and false positives carry different implications. Together, these metrics provide a comprehensive evaluation of the model's predictive performance, facilitating comparisons with other modeling approaches in the study (Rainio et al., 2024).

An experimental design process was used, involving a structured approach to ensure high internal validity. Experimental study designs follow a set of established rules and techniques specifically created for conducting scientific research.

### Model interpretability

The interpretability process for a stacked ensemble model with multiple base learners focuses on understanding the contributions of individual models and how their predictions are integrated by the meta-learner. A crucial component of this interpretative framework is the application of SHAP (Shapley Additive Explanations) values, particularly for the best-performing model within the ensemble. By utilizing SHAP values, researchers can gain insights into the specific contributions of each feature to the predictions made by this top-performing model. This method quantifies the importance of features while accounting for interactions, thus allowing for the identification of influential variables that drive the model's decisions. This analysis provides a clear understanding of the decision-making processes within the best-performing learner, laying a solid groundwork for interpreting the ensemble's overall effectiveness.

SHAP values can be employed at this level to elucidate how much the top-performing model's predictions contribute to the overall ensemble output, providing insights into the dynamics of the stacked model. While SHAP serves as the primary interpretability tool, additional methods such as integrated gradients and permutation feature importance can complement this analysis by offering broader perspectives on feature influence across the ensemble. Local interpretability methods, like LIME (Local Interpretable Model-agnostic Explanations), can further clarify individual predictions by approximating the model's behavior around specific instances. By emphasizing SHAP values for the best-performing model within this interpretative framework, the study enhances the transparency and trustworthiness of the stacked ensemble model's predictive capabilities, facilitating a comprehensive understanding of the underlying mechanisms driving its decisions (Baptista et al., 2022).

### Ethical approval

This study utilized secondary data obtained from the Demographic and Health Surveys (DHS), which are publicly available datasets. As this research involved only anonymized data with no direct interaction with participants, further ethical review was not required. All analyses were conducted in compliance with ethical standards for research involving secondary data.

## Results

### Baseline characteristic

After the DHS data from the 10 East African countries was cleaned and merged, there were a total of 61,714 instances. Of those, 9,206 (14.9%) failed to complete (had dropped out of) the Penta3 vaccination. Among the 52,508 children who completed their vaccination, 75.2% of those living in rural areas completed the vaccination compared to 24.8% in urban areas. Children whose mothers had no education had a lower completion rate (18.1%) and a higher dropout rate (25.3%) compared to those with primary education (49.5% completed, 47.8% dropped out). Wealthier families had a higher vaccination completion rate (37.2%) compared to poorer households, where 43.7% of children dropped out. Mothers whose babies were delivered at government health facilities had a much higher vaccination completion rate (78.3%) compared to home deliveries (17.0%) (Supplementary Table 2).

**Figure 2:**
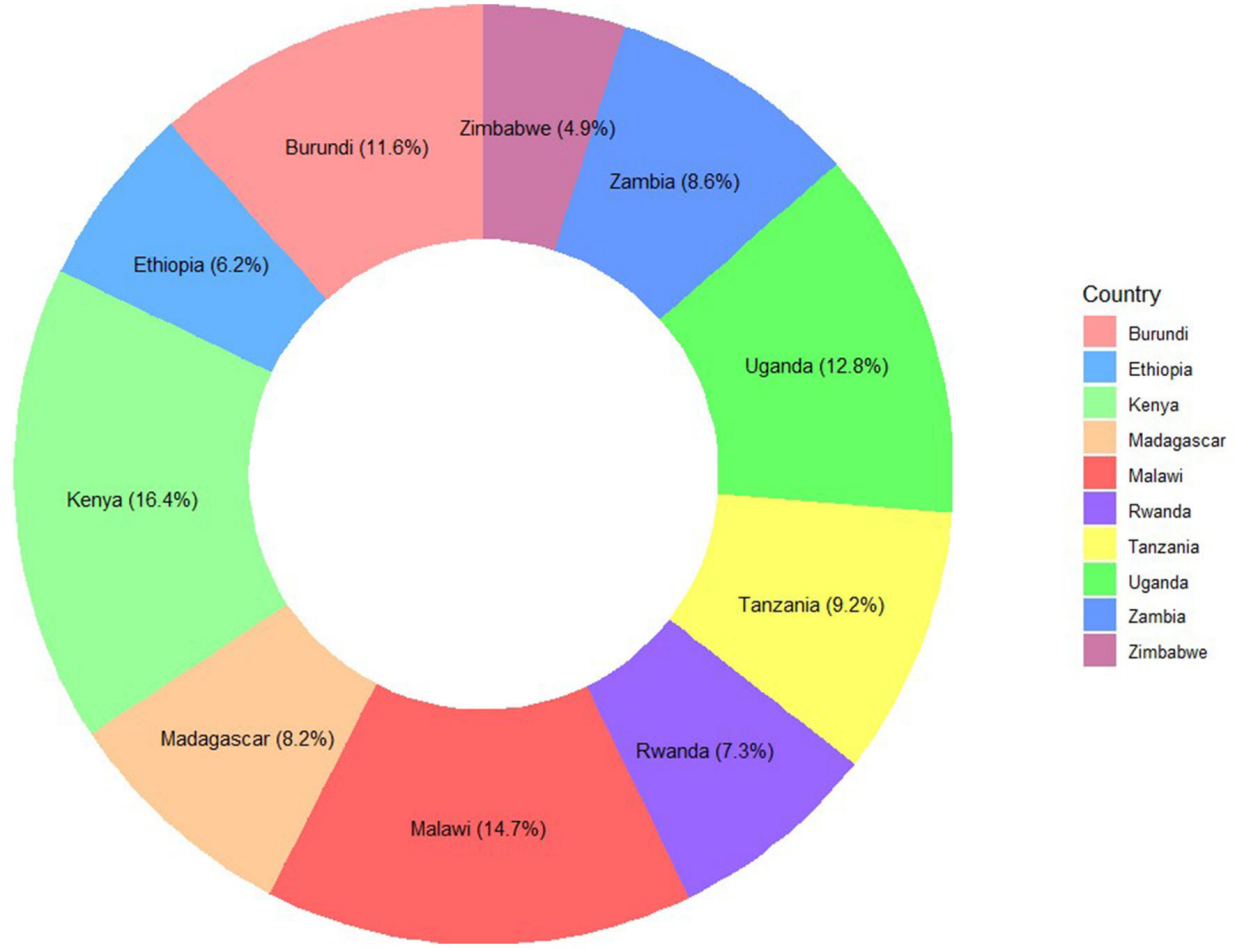
Distribution of samples from each East-African Country: Penta3 vaccination dropout prediction.

Furthermore, children whose mothers were aged 20–29 had the highest Penta3 vaccination completion rates, with 26.2% for those aged 20–24 and 25.6% for those aged 25–29. The dropout rates were also higher in these age groups, at 28.2% and 24.1%, respectively. Among countries, Kenya had the highest completion rate at 16.3%, while Uganda showed the highest dropout rate at 18.9%. Moreover, mothers with higher education levels (secondary and above) had notably better completion rates (26.7% and 5.6%, respectively), reflecting a clear association between maternal education and vaccination completion (Figure 2 and Supplementary Figure 1).

### Data preprocessing results

The dataset used in this study comprised a range of socio-economic, demographic, and health-related variables, which were initially subjected to extensive preprocessing steps to ensure suitability for machine learning modeling. Given the varied nature of the data, several stages of preprocessing were carried out to handle missing values, and categorical features. This was crucial to improve the quality of the data and ensure that it could effectively be used to build the stacked ensemble model.

First, the dataset was extracted from the DHS (Demographic and Health Survey) database, encompassing data from multiple East African countries. Two features (i.e., husband's educational status and husband's occupational status) had low levels of missing data, with 3.7% and 2.9%, respectively, while husband's age had a moderate level of missingness at 9.7%. Consequently, mode imputation was applied to the first two features. The most frequent category (mode) for each feature was identified and used to replace the missing values. For the feature “husband's age,” the pattern of missingness was analyzed and found to be Missing at Random (MAR). Given this missingness pattern, the recommended model-based imputation method (i.e., KNN) was performed using the KNN function of the VIM package. This ensured that the dataset retained as much information as possible without introducing distortions caused by missing data.

Once the missing values were addressed, feature engineering was performed. Derived features were created to capture complex interactions between variables. The media exposure variable was constructed by integrating the frequency of radio listening, newspaper reading, television viewing, and internet usage. The healthcare access feature was created based on whether the mother faces challenges in obtaining permission from her husband to visit a hospital, securing the funds required for treatment, overcoming the distance to the healthcare facility, or if attending the facility alone poses a difficulty.

### Experimental results and comparisons

We performed different experiments and comparisons for both Boruta and LASSO feature selection methods with performance metrics obtained from Random Search HPO and Grid Search HPO.

#### Experiment I: feature selection

**Figure 3:**
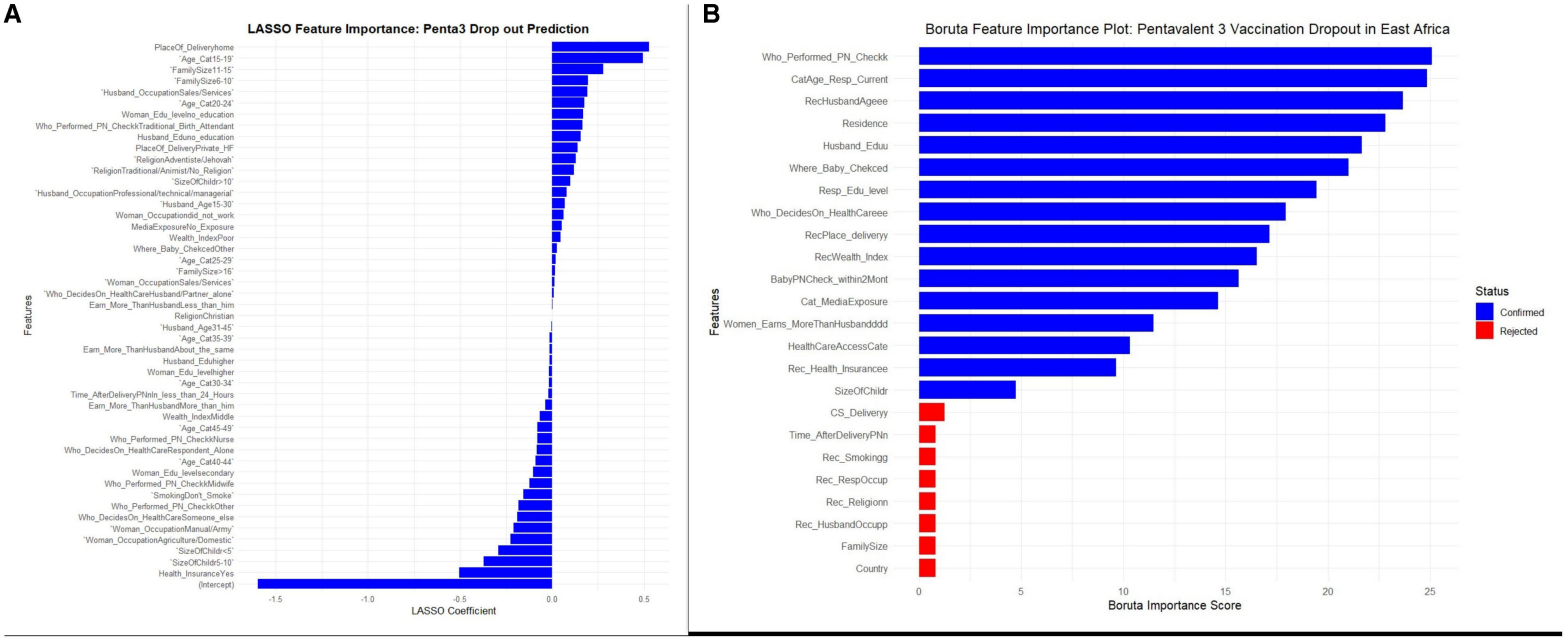
**(A, B)** LASSO and Boruta feature selections for a stacked ensemble machine learning model for the prediction of pentavalent 3 vaccination dropout in East Africa.

For experimentation purposes, both the LASSO (Least Absolute Shrinkage and Selection Operator) and the Boruta algorithm were used to identify and select the most relevant features from the dataset. For LASSO, since it requires numeric features, a matrix of encoded features and the target variable was constructed from the data, with the intercept column removed. Cross-validation was used to train the LASSO model and determine the optimal lambda (regularization parameter). The final LASSO model, fitted with the optimal lambda, identified important features with non-zero coefficients, while 26 features were eliminated. Features with both positive (direct relationship) and negative (inverse relationship) LASSO coefficients were considered important, as displayed in Figure 3A.

Unlike LASSO, which operates strictly on numerical features, Boruta does not require one-hot encoding for categorical variables. Therefore, it was performed before encoding to keep the feature space manageable and preserve the original relationships. As a result, the Boruta algorithm deemed eight features irrelevant and rejected them from further analysis. This process reduced the total number of features from 24 to 16. The results of the feature selection process by Boruta, including the importance of each variable and the rejection of the eight aforementioned features, are presented in Figure 3B.

Before model building, all categorical features deemed important by Boruta were transformed using one-hot encoding, while ordinal encoding was applied to “Educational Status of the Mother and Husband” to facilitate integration into machine learning models, which typically require numerical input. This transformation increased the number of features in the dataset from 16 to 63. Subsequently, highly correlated features were identified and removed using the “findCorrelation” function, which further reduced the number of features to 51. These preprocessing steps ensured that the categorical data were effectively utilized, enhancing model performance without compromising generalizability. The correlation matrix is illustrated on Supplementary Figure 2.

#### Experiment II: model building and optimal hyperparameters

##### Model building

To build the models, the dataset was divided into a training set comprising 70% of the data (43,199 instances) and a test set comprising 30% (18,515 instances). Class imbalance was a significant concern, as the dropout rate from the Penta3 vaccine fell well below the threshold for a balanced dataset, where the minority class constitutes < 30%−40% of the total. The imbalance ratio in the dataset was 5.71:1, indicating that for every instance of a child who dropped out (class 1), there were ~5.71 instances of children who completed their vaccination (class 0). This imbalance posed a risk of biased predictions favoring the majority class. To mitigate this issue, the Synthetic Minority Over-sampling Technique (SMOTE) was exclusively applied to the training set to generate synthetic samples for the minority class (children who dropped out of the Penta3 vaccination), thereby ensuring that no data leakage occurred during model evaluation. The SMOTE parameters, including the number of nearest neighbors (K = 5) and the duplication size (dup_size = 0), were set to their default values in the SMOTE function.

This technique effectively balanced the dataset, ensuring that the predictive models will not be biased toward the majority class while preserving the characteristics of the minority class and minimizing the risk of overfitting. The distribution of the target variable before and after the application of SMOTE is presented in Supplementary Figure 3.

Seven base learners were trained: Generalized Linear Model (GLM), Naive Bayes (NB), Decision Tree (DT), XGBoost (XGB), Random Forest (RF), Gradient Boosting Machine (GBM), and Deep Learning (DL). Each model was individually optimized using 10-fold cross-validation to prevent overfitting and ensure generalizability. The cross-validation was performed using stratified folds for all models except the GLM, which used modulo fold assignment. This approach preserved class balance across folds and ensured that the models generalized well to unseen data.

After training the base learners, their predictions were combined using a meta-learner in a stacked ensemble approach. Three different meta-learners were tested: XGBoost, Random Forest, and GLM. For each stacked ensemble, out-of-fold predictions from the base learners were used as features for the meta-learner, preventing data leakage and ensuring robust performance.

As the stacked ensemble model inherits the nfolds from base learners, a stacked cross-validation was, by default, employed for the meta-learner training. Each base learner was trained on 90% of the training data and generated out-of-fold predictions for the remaining 10%. These out-of-fold predictions were then passed to the meta-learner for training. Since the meta-learner was trained only on predictions generated from data that were held out from the base learners' training process, this prevents any form of information leakage. The key fact here is that the meta-learner did not have access to the raw features or base learner predictions from the same folds during training. This cross-validation process was repeated for all folds, ensuring that for each instance in the dataset, the corresponding meta-learner prediction was based solely on out-of-fold base learner predictions, which preserves the integrity of the model evaluation and avoids overfitting. Furthermore, early stopping was implemented in models like XGBoost, Gradient Boosting Machine (GBM), Random Forest, and Deep Learning to halt training when the performance did not improve for 50 rounds. AUC was used as a stopping metric.

##### Random search hyperparameters

The same systematic random search hyperparameter values were used for both experiments with Boruta and LASSO. The Generalized Linear Model (GLM) was trained with L2 regularization (alpha = 0.1) and the “binomial” family for binary classification. The glm function of h2o is a general framework for Generalized Linear Models (GLMs), and configuring it with the “binomial” family specifies logistic regression for binary outcomes. A single decision tree was trained with ntrees = 1, a maximum depth of 30, and min_rows = 1. XGBoost was configured with 1,000 trees, a learning rate of 0.05, and a maximum depth of 3, with early stopping after 50 rounds. The Random Forest model used 500 trees, a maximum depth of 20, and a sample rate of 0.8, while the GBM was set to 500 trees, a learning rate of 0.01, a maximum depth of 7, and early stopping after 50 rounds based on AUC. Lastly, the deep learning model had two hidden layers of 100 nodes each and was trained for 10 epochs, with early stopping after 50 rounds based on AUC. This model with two hidden layers and complex configuration fits the general definition of deep learning, which involves models with more than one layer of transformation (Table 1).

**Table 1:** Random search HPO values for Penta-3 vaccination dropout prediction.

**Base learner**	**Hyperparameters**	**Values**
Decision tree	ntrees, max_depth, min_rows	1, 30, 1
XGBoost	ntrees, learn_rate, max_depth, stopping_rounds	1,000, 0.05, 3, 50
Random forest	ntrees, max_depth, sample_rate, min_rows	500, 20, 0.8, 1
GBM	ntrees, learn_rate, max_depth, min_rows, stopping_rounds	500, 0.01, 7, 5, 50
Deep learning	hidden, epochs, stopping_rounds	(100, 100), 10, 50

#### Experiment III: performance metrics with Boruta FS for both HPO methods

Table 2 presents the performance metrics for seven base learners and three Stacked Ensemble Models (SEMs) using the Boruta Feature Selection method under two hyperparameter optimization (HPO) approaches, Grid Search and Random Search.

**Table 2:** Performance metrics of Boruta FS with both random search and grid search HPOs: Penta 3 dropout prediction.

**Model**	**HPO method**	**AUC**	**Accuracy**	**Precision**	**Recall**	**F1-score**
XGBoost	Grid search	0.824	0.826	0.821	0.805	0.809
	Random search	0.813	0.815	0.814	0.798	0.799
GBM	Grid search	0.836	0.834	0.835	0.811	0.819
	Random search	0.822	0.824	0.826	0.806	0.809
Random forest	Grid search	0.814	0.817	0.823	0.804	0.809
	Random search	0.801	0.803	0.805	0.782	0.789
Naive Bayes	Grid search	0.697	0.685	0.717	0.699	0.699
	Random search	0.672	0.637	0.757	0.678	0.707
Logistic regression	Grid search	0.707	0.696	0.702	0.636	0.663
	Random search	0.685	0.673	0.786	0.754	0.764
Deep learning	Grid search	0.738	0.704	0.787	0.632	0.697
	Random search	0.705	0.685	0.752	0.621	0.678
Decision tree	Grid search	0.774	0.756	0.714	0.685	0.694
	Random search	0.753	0.713	0.705	0.658	0.674
SEM XGB meta	Grid search	0.807	0.784	0.827	0.787	0.799
	Random search	0.798	0.766	0.792	0.824	0.804
SEM RF meta	Grid search	0.872	0.825	0.852	0.772	0.808
	Random search	0.861	0.813	0.834	0.764	0.793
SEM GLM meta	Grid search	0.832	0.793	0.796	0.747	0.764
	Random search	0.814	0.771	0.818	0.727	0.762

Among the Stacked Ensemble Models (SEMs), the Random Forest-based ensemble using Grid Search delivers an outstanding AUC of 0.872 with a high Accuracy of 0.825, making it the top performer overall. The XGBoost-based ensemble also performs well, achieving a solid F1-score of 0.804 with Random Search, showcasing its balanced precision and recall. Additionally, the GLM-based ensemble using Grid Search shows competitive results, with a respectable AUC of 0.832 and an F1-score of 0.764, demonstrating its reliability as a meta-learner. These results highlight the effectiveness of different meta-learners in stacked ensemble models, with Random Forest-based ensembles standing out as the most robust (Table 2).

#### Experiment IV: performance metrics with LASSO FS for both HPO methods

##### Performance metrics of LASSO FS with random search HPO

**Figure 4:**
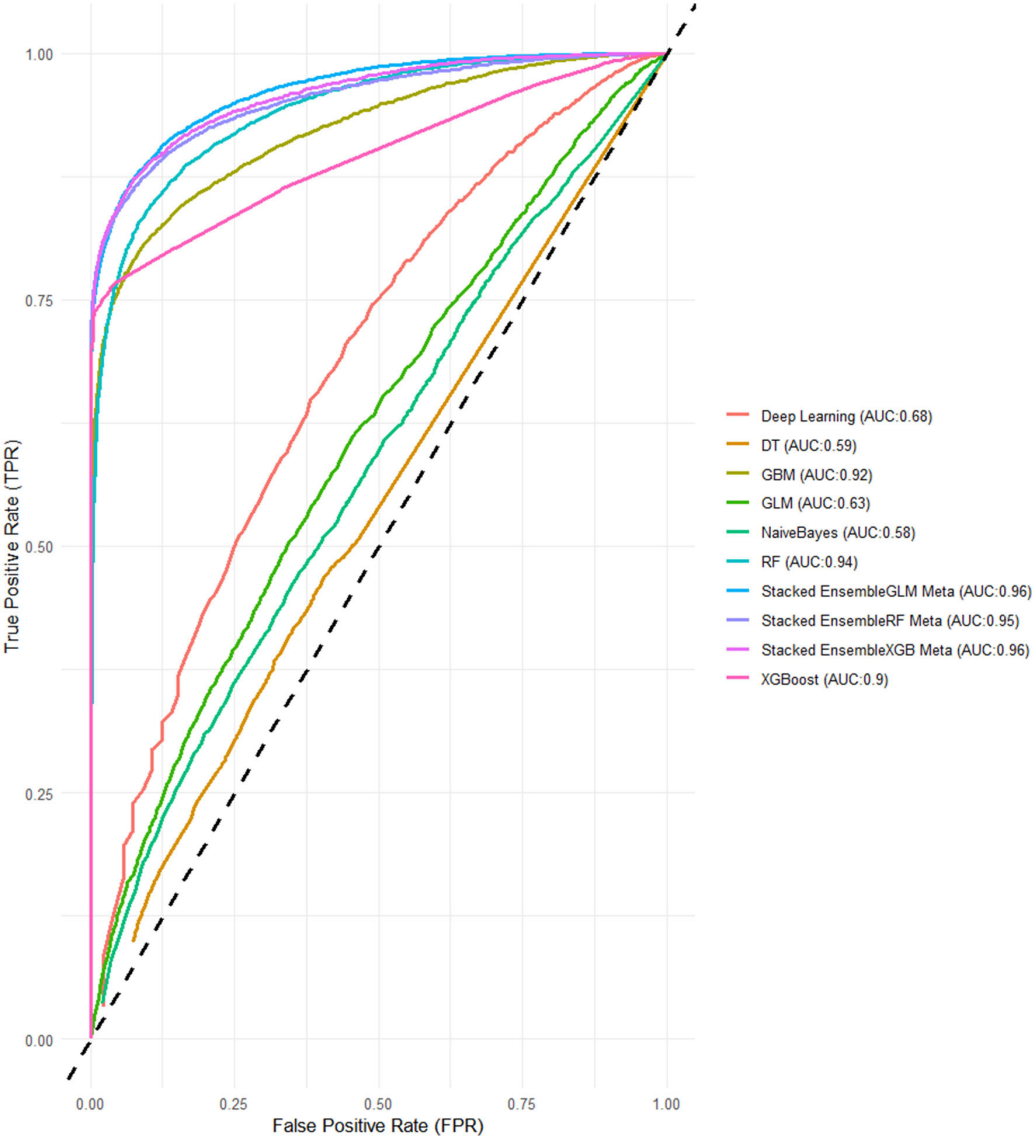
ROC-AUCs of LASSO FS with and random search HPO: Penta 3 dropout prediction in East-Africa.

For the models using LASSO feature selection with Random Search hyperparameter optimization, the XGBoost-based ensemble achieves the highest overall performance, with an AUC of 0.961 and an impressive Accuracy of 0.901, highlighting its strong predictive capability. Similarly, the Random Forest-based ensemble also performs exceptionally well, with an AUC of 0.953 and an Accuracy of 0.899. Meanwhile, the GLM-based ensemble shows consistent results, with an AUC of 0.957 and an Accuracy of 0.902, proving to be a competitive option. These stacked ensembles consistently outperform the individual base learners across most metrics, particularly in terms of AUC and Accuracy (Figure 4 and Table 3).

**Table 3:** Performance metrics of LASSO FS with random search HPO: Penta 3 dropout prediction.

**Model**	**Accuracy**	**Precision**	**Recall**	**F1 score**
NB	0.504	0.484	0.937	0.638
DT	0.774	0.736	0.798	0.766
GBM	0.860	0.886	0.804	0.843
RF	0.810	0.807	0.780	0.793
DL	0.552	0.517	0.930	0.664
XGB	0.870	0.949	0.763	0.846
GLM	0.514	0.489	0.947	0.645
SEM XGB meta	0.901	0.922	0.860	0.890
SEM RF meta	0.899	0.939	0.839	0.886
SEM GLM meta	0.902	0.918	0.866	0.892

##### Performance metrics of LASSO FS with grid search HPO

The grid search for XGBoost evaluated 108 models, with the best model achieved using a learning rate of 0.1, max depth of 9, 200 trees, 3 minimum rows, and a sample rate of 0.8. The GBM grid search evaluated 110 models, and the best combination was obtained with a learning rate of 0.1, max depth of 10, 26 trees, 5 minimum rows, and a sample rate of 0.8. The RF grid search evaluated 181 models, with the top-performing models using a max depth of 30, 13 trees, 1 minimum row, and a sample rate of 0.8. The NB grid search, with only 3 models evaluated, showed similar performance with Laplace smoothing values of 0, 1, and 2. The GLM grid search evaluated 16 models, and the best combination was alpha = 0.0 and lambda = 0.001, with minimal variations in performance across other similar values. The deep learning grid search evaluated multiple configurations, and the best model was achieved with hidden layers of (100, 100), 10 epochs, and a learning rate of 0.01. Finally, the Decision Tree grid search identified the optimal model with a max depth of 10, 5 minimum rows, and a sample rate of 0.8 (Table 4).

**Table 4:** Hyperparameter tuning results: grid search HPO parameter ranges and optimal values for Penta-3 vaccination dropout prediction.

**Model name**	**Hyperparameter**	**Range**	**Default value (*R*)**	**Optimal value**
XGBoost	ntrees	c(100, 200, 400, 600, 800, 1,000)	100	200
	learn_rate	c(0.01, 0.05, 0.1)	0.3	0.1
	max_depth	c(3, 6, 9, 12)	6	9
	min_rows	c(3, 5, 10, 13, 15)	10	3
	sample_rate	c(0.7, 0.8, 0.9)	1	0.8
GBM	ntrees	c(100, 200)	100	260
	learn_rate	c(0.01, 0.05, 0.1)	0.1	0.1
	max_depth	c(5, 10, 15)	6	10
	min_rows	c(3, 5, 10)	10	5
	sample_rate	c(0.8, 0.9)	1	0.8
Random forest	ntrees	c(50, 100, 200)	500	100
	max_depth	c(10, 20, 30)	20	30
	min_rows	c(1, 5, 10)	1	1
	sample_rate	c(0.7, 0.8, 0.9)	1	0.8
Naive Bayes	laplace	c(0, 1, 2)	1	1
	use_all_factor_levels	c(TRUE, FALSE)	FALSE	TRUE
Logistic regression (GLM)	alpha	c(0, 0.1, 0.5, 1)	0.5	0
	lambda	c(0, 0.001, 0.01, 0.1)	0	0.001
Deep learning	hidden	c(50, 50), c(100, 100), c(50, 100, 50)	c(50, 50)	c(100, 100)
	epochs	c(10, 20, 50)	10	10
	learning_rate	c(0.01, 0.05, 0.1)	0.01	0.01
	activation	c(“Rectifier,” “Tanh,” “Maxout”)	“Rectifier”	“Rectifier”
Decision tree	max_depth	c(5, 10, 15)	30	10
	min_rows	c(1, 5, 10)	20	5
	sample_rate	c(0.7, 0.8, 0.9)	1	0.8

**Figure 5:**
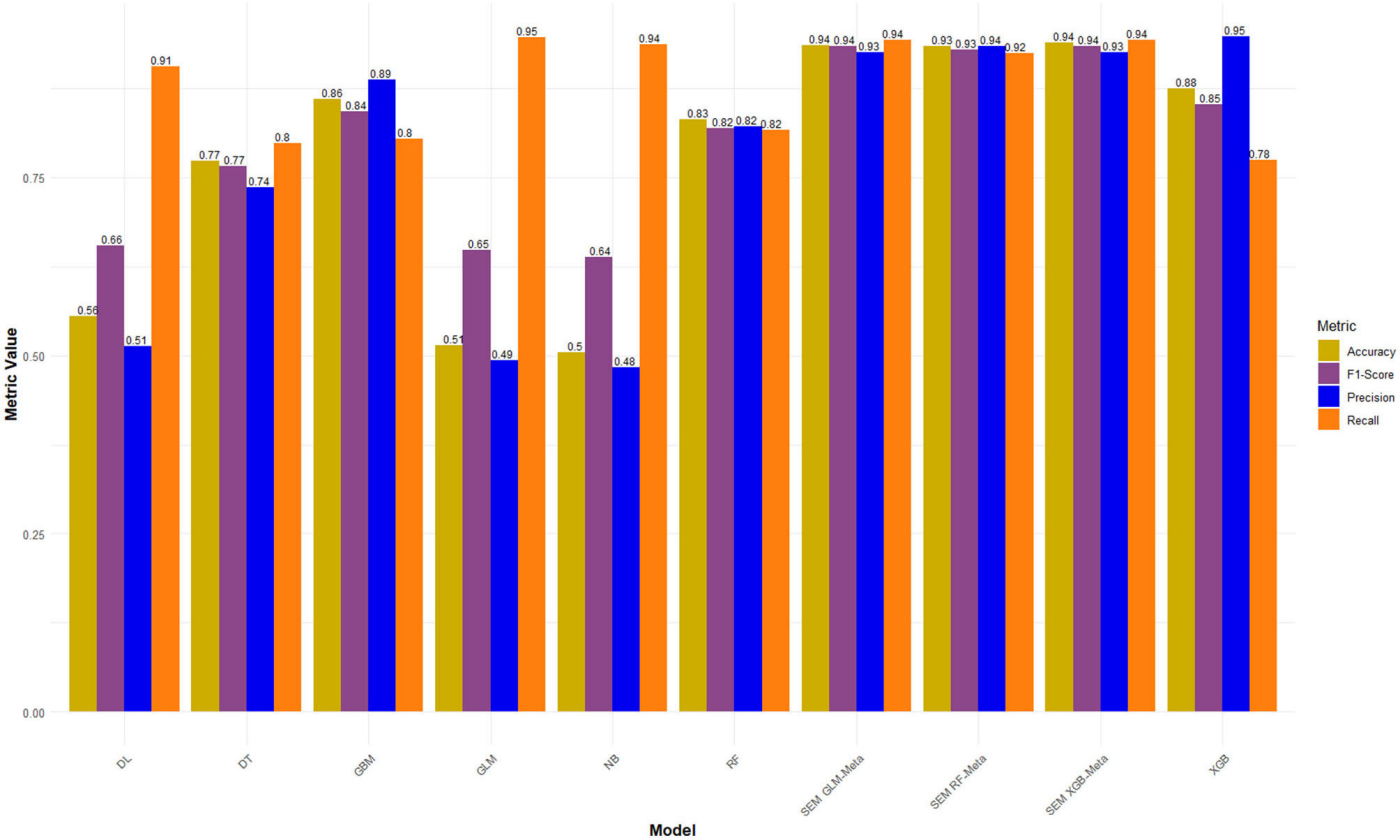
Performance metrics of LASSO FS with and grid search HPO: Penta 3 dropout prediction in East-Africa.

**Figure 6:**
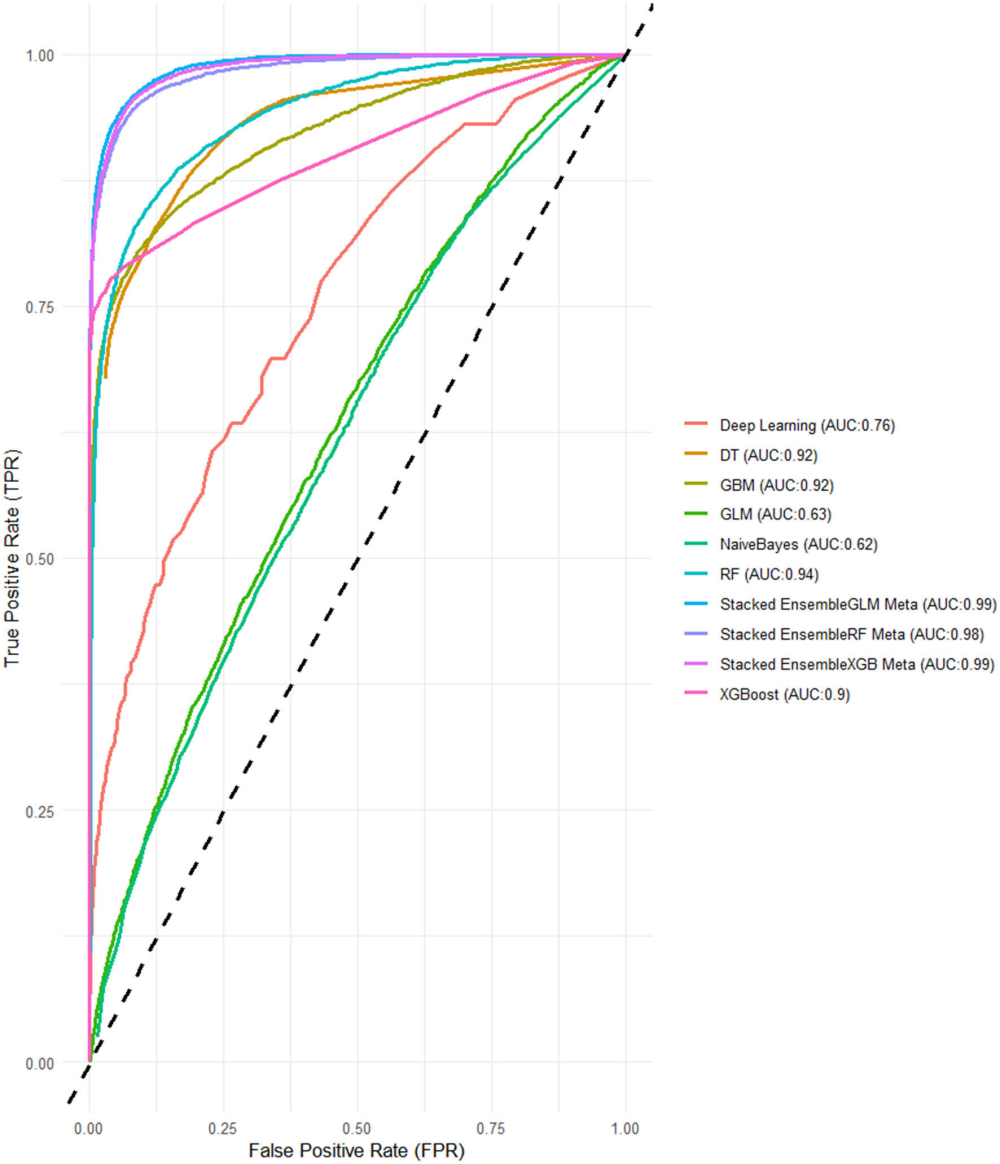
ROC-AUCs of LASSO FS with and grid search HPO: Penta 3 dropout prediction in East-Africa.

In this final experiment using LASSO feature selection with Grid Search HPO, the XGBoost-based ensemble stands out once again with the highest AUC of 0.994 and a strong Accuracy of 0.939, demonstrating its consistent performance across experiments. The GLM-based ensemble matches its AUC of 0.990, along with a comparable Accuracy 0.936, proving its reliability and competitiveness. Meanwhile, the Random Forest-based ensemble follows closely with an AUC of 0.983 and an Accuracy of 0.935 (Figures 5, 6).

When compared to the previous experiments, both feature selection methods, Boruta and LASSO, yielded strong results, particularly for the stacked ensembles, which consistently outperformed the individual base learners. The Grid Search HPO method, especially in this LASSO feature selection experiment, delivered slightly better overall performance than Random Search, with noticeably higher AUC scores across all SEMs. Notably, the XGBoost and GLM-based ensembles achieved perfect alignment in key metrics here, while in previous experiments, XGBoost ensembles generally held a slight edge (Figures 5, 6).

These final results confirm that stacked ensemble models, especially those using Random Forest and XGBoost as meta-learners, deliver robust and accurate predictions, with LASSO feature selection combined with Grid Search yielding the strongest overall performance across all experiments.

Due to its overall superior performance, the XGBoost Meta-Learner Stacked Ensemble, built with LASSO-selected features and optimized through Grid Search, was selected for further analysis using advanced interpretability tools like SHAP (SHapley Additive exPlanations) bar and beeswarm plots, providing deeper insights into feature contributions and model behavior. Overall, these results demonstrate that the stacked ensembles effectively leveraged the strengths of the base learners, leading to a significant improvement in predictive performance.

### Model interpretability

The output of the SHAP (SHapley Additive exPlanations) values reveals intricate insights into the attributes influencing predictions made by the best-performing Stacked Ensemble XGB-Meta learner Model. This SHAP matrix highlights the contributions of each feature to the model's output.

In analyzing the SHAP BeeSwarm plot for model interpretability, several nuances must be considered to avoid misinterpretation. Features with a mix of colors where both high (golden) and low (purple) feature values have SHAP values that vary between positive and negative impacts, suggest the presence of non-linear relationships or interactions between features. In such cases, high values of the feature can contribute positively to the model's predictions for some instances, while contributing negatively for others, indicating that the feature's influence is context-dependent. From a practical decision-making perspective, this means that interventions or policies relying on these features must be carefully tailored to specific subgroups or conditions. For example, a feature might be highly beneficial for one group but could have adverse effects or little relevance in another, suggesting the need for personalized or context-aware strategies.

Additionally, features for which data points (instances) predominantly cluster around the SHAP value of 0, with minimal horizontal spread, indicate that these features have little to no impact on most predictions. These features may be irrelevant or redundant for the model's decision-making process, though they might occasionally have a significant effect in a small subset of instances. Careful attention to such patterns ensures that only meaningful features are prioritized for interpretation or model refinement, avoiding overemphasis on features with low or highly conditional importance (Mane et al., 2024; Lundberg et al., 2020).

In terms of research direction, these findings point to areas where further investigation is needed to understand the contexts or subpopulations where these features hold more weight. This could guide future studies to explore the non-linear relationships or interactions identified through the SHAP analysis, ultimately informing both model refinement and the development of more targeted, effective interventions or policies.

Considering these facts, seven features (place of delivery, health insurance, healthcare access, type of health professional who performed the post-natal check-up, wealth index, and who decides on the healthcare need of the family) visualized in the SHAP BeeSwarm and bar plots were identified as having significant and interpretable contributions to the model's predictions. These features exhibited considerable SHAP value variability and relatively clear patterns of influence, highlighting their importance in driving the model's decisions. In contrast, the remaining features, characterized by either tight clustering around the zero SHAP value or a lack of clear directional impact, were deemed less influential. Their mixed and limited spread suggests that these features contribute minimally to the model's predictive power or have contextually dependent effects. This understanding directs the focus toward the most relevant features, thereby enhancing the interpretability and reliability of the model.

**Figure 7:**
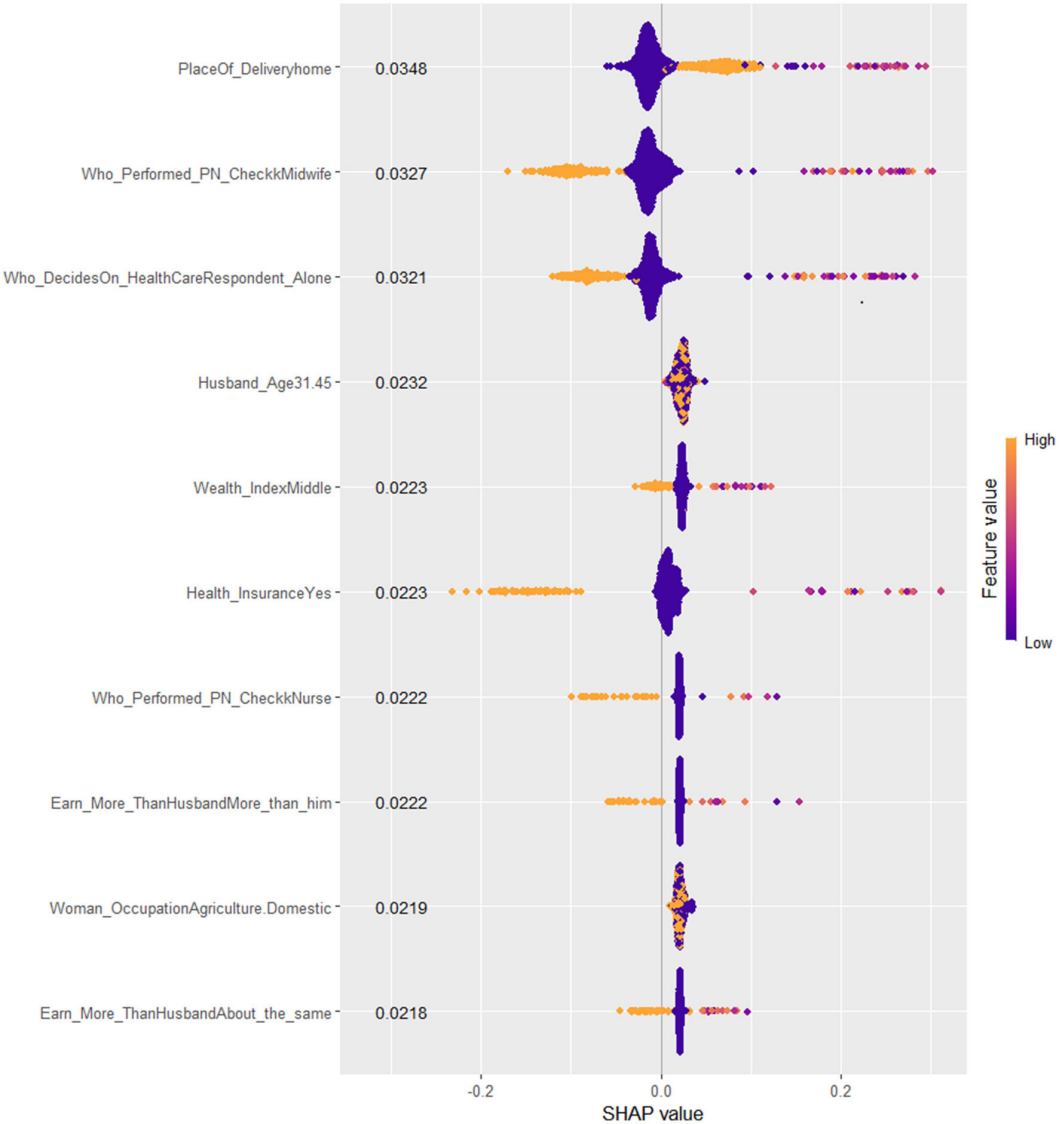
SHAP Beeswarm plot of the best XGB-meta stacked ensemble model for Penta 3 vaccination dropout prediction.

**Figure 8:**
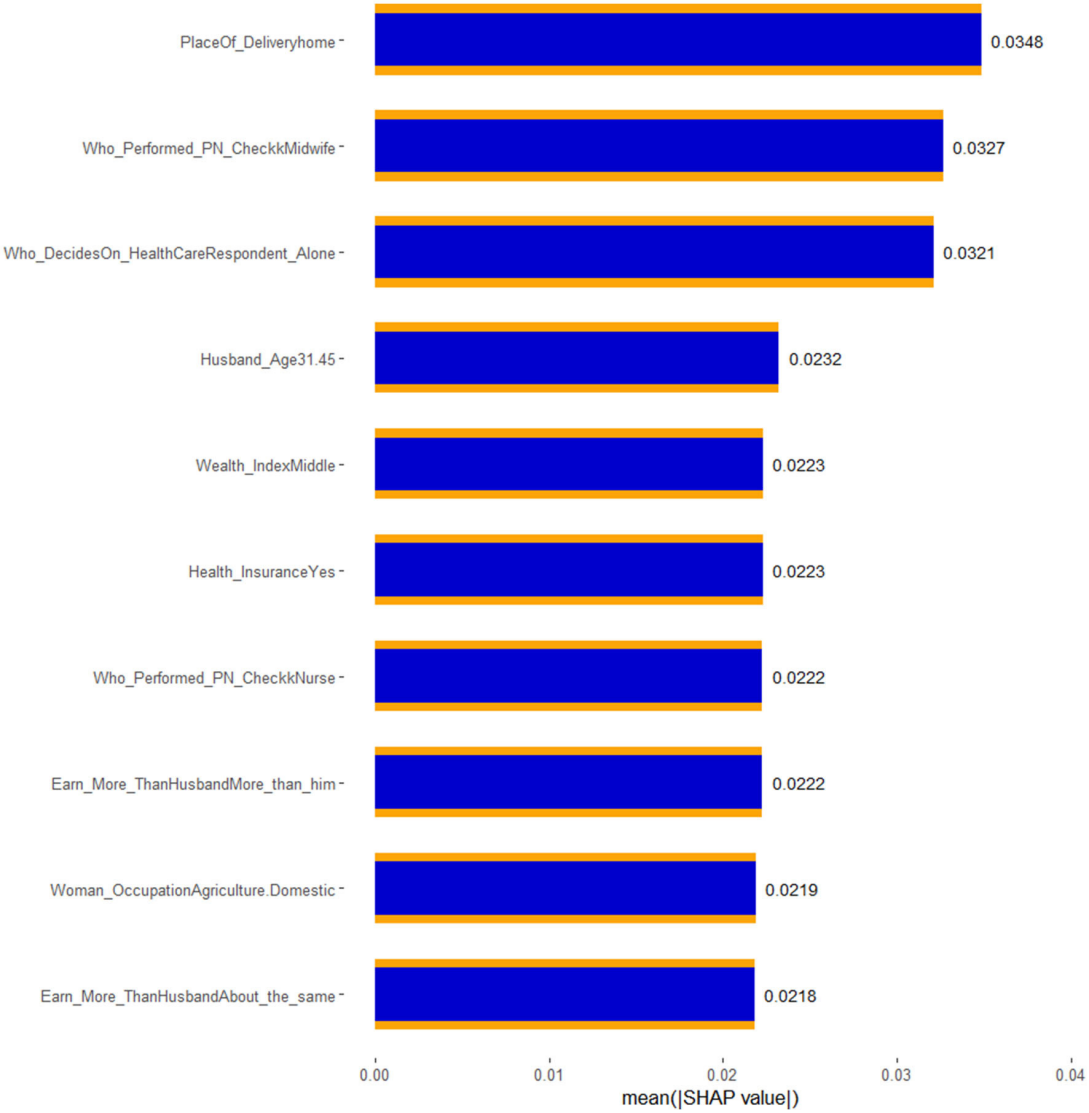
SHAP bar plot of the best XGB-meta stacked ensemble model for Penta 3 vaccination dropout prediction.

Accordingly, the best-performing stacked ensemble model predicted that children born at home have a substantial positive impact, with a SHAP value of 0.0348, indicating that home births are associated with a higher probability of Penta 3 vaccination dropout. Similarly, children of women who earns more money than the husband/father and who earns about the same amount of money exhibited notable negative impacts (SHAP value: −0.0222 and −0.0218, respectively) on Penta 3 vaccination dropout. In contrast, children of mothers with health insurance showed a negative impact, with a SHAP value of −0.0223, suggesting a lower likelihood of dropout. The SHAP values for decision on the healthcare need of the family indicate that when the child's mother decides, the likelihood of Penta 3 vaccination dropout slightly decreases (SHAP value: 0.0321). Post-natal check-ups conducted by midwifery professionals had a negative impact on dropout, suggesting that mothers who received post-natal care from these professionals have a reduced likelihood of dropout (SHAP value: −0.0327). Middle wealth index families also contributed negatively to the prediction, indicating a lower probability of Penta 3 vaccination dropout (SHAP value: −0.0223). Overall, this detailed analysis of SHAP values not only clarifies which features are driving the model's predictions but also underscores the complex interplay between socioeconomic factors, healthcare access, and demographic characteristics in determining health-related outcomes (Figures 7, 8).

## Discussion

This study aimed to develop and evaluate a stacked ensemble machine learning models that can accurately predict pentavalent 3 vaccination dropout in East Africa using the Demographic and Health Surveys (DHS) dataset. The DHS provides rich, nationally representative data on health, population, and nutrition, making it an ideal source for analyzing health outcomes across diverse populations.

We evaluated various machine learning models and stacked ensemble methods using Boruta and LASSO feature selection techniques combined with Random Search and Grid Search hyperparameter optimization. The results demonstrated that Stacked Ensemble Models (SEMs) consistently outperformed individual base learners across key metrics. In Boruta feature selection experiments, the Random Forest-based SEM achieved strong performance, showcasing the significant improvement that stacked ensembles bring by combining the strengths of multiple base models.

In the LASSO feature selection experiments, the XGBoost Meta-Learner Stacked Ensemble optimized with Grid Search emerged as the top performer, consistently demonstrating high AUC, Accuracy, and F1-score. This model outperformed other base learners and SEMs, underscoring the effectiveness of XGBoost as a meta-learner in stacking. The Random Forest-based SEM and GLM-based SEM also delivered strong results, further confirming the power of stacking diverse models for improved predictive performance.

The final XGBoost Meta-Learner Stacked Ensemble, built with LASSO-selected features and optimized through Grid Search, was selected for further analysis using advanced interpretability tools like SHAP bar and beeswarm plots. These tools provided deeper insights into feature contributions and model behavior. Overall, the experiments highlight the superiority of stacked ensembles, particularly when using Grid Search for optimization and LASSO for feature selection, making the XGBoost-based SEM the optimal choice for complex machine learning tasks.

By interpreting the SHAP values of the best stacked ensemble model, we were able to assess the contribution of individual features to the model's predictions, offering transparent and interpretable insights into the drivers of vaccination dropout. Seven key features were identified as critical in explaining dropout rates: place of delivery, health insurance, healthcare access, type of health professional who performed the post-natal check-up, wealth index, and who decides on the healthcare need of the family. These findings provide valuable insights into the complex interplay of socioeconomic and healthcare-related factors influencing vaccination outcomes in East Africa, where access to healthcare remains a significant challenge.

The place of delivery emerged as a key determinant, with the model revealing a substantial positive SHAP value of 0.0348 for home births, indicating a strong association with higher Penta 3 vaccination dropout in East Africa. This finding reflects the significant challenges that arise when children are born at home, particularly in rural or underserved areas where access to healthcare services is limited. In many East African countries, home births are common due to cultural practices, geographic isolation, and lack of healthcare facilities, leading to missed opportunities for postnatal care and immunization follow-up. The absence of skilled birth attendants in home settings likely contributes to the higher dropout rates observed, as mothers may not receive timely reminders or guidance on vaccination schedules (Ntenda et al., 2022; Odiit and Amuge, 2003).

Alternative explanations for this finding could include the broader socioeconomic conditions of households opting for home births. These families may face other barriers, such as limited transportation, financial constraints, or inadequate health literacy, which further exacerbate the risk of vaccination dropout. While the SHAP value indicates a strong association between home births and dropout, it is essential to recognize that causality cannot be directly inferred. Furthermore, cultural preferences for home births, especially in rural and remote communities, may limit the effectiveness of interventions focused solely on healthcare access. Future policies should consider integrating traditional birth attendants with formal healthcare systems to ensure that newborns receive timely vaccinations, even in home birth scenarios (Mmanga et al., 2022).

The study found that the presence of health insurance was associated with a negative SHAP value of −0.0223, suggesting that children of mothers with health insurance were less likely to experience Penta 3 vaccination dropout. In East Africa, health insurance coverage remains low, but where available, it often facilitates better access to healthcare services, reducing the financial burden of medical visits and vaccinations. Mothers with insurance are more likely to visit healthcare facilities regularly, leading to better adherence to vaccination schedules. This protective effect of health insurance aligns with findings from other low- and middle-income countries, where health insurance has been shown to improve healthcare utilization and child health outcomes (Fenta et al., 2023; Smith et al., 2006).

However, the effect of health insurance might differ across countries in the region, given the varying structures and coverage of health insurance schemes. In some cases, public health insurance programs may be limited in scope, covering only certain services or medications, which could reduce their overall impact on vaccination adherence. Additionally, the availability of health insurance may be correlated with other favorable factors, such as higher income, urban residence, or education, which may also contribute to lower dropout rates. While the SHAP value underscores the importance of insurance, future studies should explore the specific mechanisms through which insurance coverage enhances vaccination rates in East Africa and identify strategies to expand coverage to underserved populations (Escobar et al., 2011; Kalies et al., 2008).

Mother's/woman's earning was another important features, indicating that mothers who earn more money or the same amount of money as their husband were less likely to have children who dropped out of the Penta 3 vaccination schedule. This finding is consistent with extensive research showing that maternal earning is a critical determinant of child health outcomes in sub-Saharan Africa. Financially secured mothers are generally more empowered to make healthcare decisions, have greater access to healthcare services, and are more likely to prioritize preventive measures like vaccinations for their children. Hence, their financial autonomy not only enhances their ability to cover direct and indirect healthcare costs but also strengthens their role in advocating for their children's wellbeing, reducing the likelihood of missed or delayed vaccinations (Yeboah et al., 2025; Bain et al., 2022).

Maternal financial independence often shifts household dynamics, allowing women greater influence over resource allocation, including healthcare spending. This leverage improves household nutrition, access to health information, and consistent healthcare-seeking behaviors. Women with independent earnings are also more knowledgeable about healthcare options and confident in engaging with providers, ensuring vaccination adherence. Beyond financial power, maternal earning reflects broader societal shifts in gender roles and equity. Policies supporting maternal employment and income generation are essential for enhancing empowerment and improving child health outcomes, particularly in vaccination programs (Zahidi et al., 2024; Fawole and Adeoye, 2015).

The SHAP value for decision-making on the healthcare needs of the family was 0.0321, indicating that when the mother is the primary decision-maker, the likelihood of Penta 3 vaccination dropout decreases. In many sub-Saharan African contexts, maternal involvement in healthcare decisions is a crucial factor influencing child health outcomes. Mothers who have the autonomy to make decisions regarding their children's health are more likely to prioritize preventive measures such as vaccinations. This aligns with existing research suggesting that maternal empowerment is key to improving child health indicators (Ozawa et al., 2016; Danchin et al., 2018).

However, decision-making power within the household is often complex. While maternal autonomy is important, other factors such as family dynamics, cultural norms, and paternal involvement can also influence vaccination adherence. In some cases, mothers may still face constraints despite being the primary decision-makers, including limited access to financial resources or healthcare services. Therefore, efforts to reduce vaccination dropout should not only empower mothers but also address broader socioeconomic and cultural barriers that may impact their ability to act on healthcare decisions. Policies that promote gender equity in healthcare decision-making, coupled with initiatives to increase maternal financial independence, can contribute to improving vaccination coverage in low-resource settings (Damnjanović et al., 2018).

Post-natal care provided by midwifery professionals had a SHAP value of −0.008, indicating a protective effect against Penta 3 vaccination dropout. In many East African countries, midwives play a critical role in maternal and child health, particularly in rural areas where doctors may be scarce. Midwives often provide essential care during and after childbirth, including counseling mothers on the importance of vaccinations. This finding aligns with evidence from other low-income regions, where midwifery-led care has been associated with improved maternal and child health outcomes (Charbit and Omrane, 2023; Schoeps et al., 2013).

However, the relatively modest SHAP value suggests that while midwifery care contributes to reducing dropout rates, it may need to be complemented by broader health system interventions. In some areas, midwives may lack sufficient training, resources, or support to deliver comprehensive postnatal care, which could limit their effectiveness in promoting vaccination adherence. Additionally, the integration of midwifery services with formal healthcare systems may vary across countries, affecting the consistency of care provided. Expanding midwifery training programs, enhancing community health outreach, and integrating vaccination services into postnatal care visits could further strengthen the role of midwives in reducing vaccination dropout (Frawley et al., 2020; Kaufman et al., 2019).

The wealth index, particularly for middle-income families, was associated with a negative SHAP value of −0.006, indicating that children from middle-income households had a lower likelihood of vaccination dropout. Wealthier families typically have better access to healthcare services, transportation, and information, all of which contribute to higher vaccination adherence. In East Africa, where wealth disparities are significant, middle- and upper-income families may have more consistent access to healthcare, enabling them to adhere to vaccination schedules more effectively (Shiferie et al., 2023; Khan and Saggurti, 2020).

However, it is important to note that wealth alone may not fully explain vaccination behavior. Cultural practices, health literacy, and social norms may also influence whether families follow through with vaccination appointments. In addition, wealthier families in rural areas may still face access challenges despite their financial resources, particularly if healthcare facilities are far away or poorly staffed. While the SHAP value highlights the protective role of wealth, it also suggests the need for targeted interventions aimed at lower-income families who are most at risk of vaccination dropout. Strengthening healthcare systems to provide equitable access regardless of socioeconomic status is essential to achieving higher vaccination coverage across all income groups (Adebowale et al., 2019; Hajizadeh, 2018).

In conclusion, the SHAP-based analysis of the stacked ensemble model revealed critical insights into the factors driving Penta 3 vaccination dropout in East Africa. These findings highlight the importance of addressing both socioeconomic and healthcare access barriers in improving vaccination adherence in the region. While the machine learning model provided valuable predictive insights, further research and targeted interventions are necessary to address the multifaceted challenges faced by vulnerable populations in East Africa.

### Limitations and strengths

Despite the valuable insights gained from this study, a few limitations must be acknowledged. First, the reliance on secondary data from the Demographic and Health Surveys (DHS) may restrict the depth of analysis regarding the causal relationships between the identified features and Penta 3 vaccination dropout. While DHS datasets are comprehensive and nationally representative, they are inherently cross-sectional, capturing a snapshot of health behaviors at a single point in time. This limitation constrains the ability to assess temporal dynamics or changes in vaccination behavior, which could vary significantly due to interventions, policy changes, or evolving socioeconomic conditions. Additionally, self-reported measures within the dataset may introduce biases related to recall or social desirability, particularly in sensitive areas such as healthcare access and maternal education. Such biases could obscure the true extent of the relationships between variables, potentially impacting the reliability of the findings.

On the other hand, this study benefits from several notable strengths that enhance its contribution to understanding vaccination dropout in East Africa. The use of a stacked ensemble machine learning model, particularly with the XGBoost meta-learner, allowed for improved predictive performance and interpretability compared to traditional modeling approaches. By integrating multiple base learners, the study effectively leveraged the strengths of different algorithms, mitigating the limitations inherent to individual models. Moreover, the application of SHAP values provided a robust framework for understanding feature importance, offering nuanced insights into how various socioeconomic and healthcare factors influence vaccination outcomes. The focus on a relevant and rich dataset, coupled with the application of advanced analytical techniques and comparisons, make this study as a significant addition to the literature on child health and immunization, informing policymakers and practitioners about critical intervention points to reduce vaccination dropout rates in the region.

## Conclusion and recommendation

The stacked ensemble model has elucidated key attributes contributing to Penta 3 vaccination dropout among children in East Africa. Notably, features such as place of delivery, health insurance, maternal earning, healthcare need decision maker, post-natal care by midwifery professionals, and wealth index emerged as significant predictors of vaccination adherence. These findings underscore the complex interplay between socioeconomic determinants and healthcare accessibility, revealing that children born at home and those from families with limited education and media exposure are at a higher risk of vaccination dropout. While the results provide valuable insights into the challenges of maintaining vaccination coverage, they also emphasize the necessity for targeted strategies that address these multifaceted issues to improve health outcomes in vulnerable populations.

Based on the findings of this study, it is essential to develop and implement comprehensive public health strategies aimed at increasing Penta 3 vaccination coverage in East Africa. Interventions should focus on enhancing healthcare access, particularly for families residing in rural areas where home births are prevalent. Additionally, public health campaigns should prioritize improving maternal education and media exposure, which have been shown to significantly influence vaccination rates. Strengthening the role of midwifery professionals in post-natal care could further mitigate dropout risks by providing mothers with essential information and support regarding vaccination. Finally, policymakers should consider integrating socioeconomic factors into health planning, ensuring that programs are tailored to address the specific needs of lower-income families to promote equity in healthcare access and utilization.

## Data Availability

Publicly available datasets were analyzed in this study. This data can be found here: https://www.dhsprogram.com/data.
