# Supplementary material for: A stacked ensemble machine learning model for the prediction of pentavalent 3 vaccination dropout in East Africa
Source: Front Big Data. 2025 Apr 7;8:1522578. doi: 10.3389/fdata.2025.1522578 (PMC12009798; doi:10.3389/fdata.2025.1522578)
Supplement: Supplementary file 1 [file Data_Sheet_1.PDF]

Supplementary Materials for a Stacked Ensemble ML Model Predicting Penta 3 Vaccination Dropout in East Africa

Supplementary Table 1 Features and Labels for Penta 3 Vaccination Dropout Prediction

S.No	Features	Feature Labels
1	Mother's age	Age of the mother in years (15-19, 20-24, 25-29, 30-34, 35-39, 40-44, 45-49)
2	Place of residence	Location of residence (Urban or Rural)
3	Mother's education	Mother's highest level of education attained (No education, Primary, Secondary, Higher)
4	Country	Country of residence (Tanzania, Burundi, Ethiopia, Kenya, Madagascar, Malawi, Rwanda, Uganda, Zambia, Zimbabwe)
5	Under-five children	Total number of children under five in the household (0, <5, 5-10, >10)
6	Household size	Household family size (less than 5, 6-10, 11-15, greater than 16 members)
7	Media exposure	Mother's exposure to media (No exposure, Has exposure)
8	Wealth index	Wealth index of the household (Rich, Middle, Poor)
9	Smoking	Mother's cigarette smoking habit (Does not smoke, Smokes)
10	Healthcare access	Difficulty in accessing healthcare (No problem, Big problem)
11	Mother's occupation	Mother's occupational status (Did not work, Professional/technical/managerial, Sales/services, Agriculture/domestic, Manual/army)
12	Place of delivery	Place of delivery of the baby (Home, Government Health Facility, Private Health Facility, Other)
13	Baby postnatal check	Whether the baby had a postnatal check within 2 months (Yes, No)
14	Place of baby check	Location where baby was first checked postnatally (Private Health Facility, Home, Government Health Facility, Other, Did not have check-up)
15	Time of PN check	Time when the mother received postnatal check-up after delivery (Did not have check-up, Less than 24 hours, Within a week, Greater than one week)
16	Postnatal checkup provider	Professional who performed the mother's postnatal check-up (Doctor, Midwife, Nurse, Traditional Birth Attendant, Community/Village Health Worker, Other, Did not have check-up)
17	Religion	Religion of the family (Christian, Muslim, Adventist/Jehovah, Traditional/Animist/No Religion, Other)
18	Health insurance	Whether the family is covered by health insurance (Yes, No)
19	CS delivery	Whether the baby was born via Caesarean section (Yes, No)
20	Husband's education	Husband’s highest level of education attained (No education, Primary, Secondary, Higher, Do not have husband)

21	Husband's occupation	Husband’s occupational status (Did not work, Professional/technical/managerial, Sales/services, Agriculture/domestic, Manual/army, Do not have husband)
22	Husband's age	Age of the husband (15-30, 31-45, 46-60, Over 61, Do not have husband)
23	Mother's earning	Mother's earnings compared to her husband's (More than him, Less than him, About the same, Do not have husband/Living with partner)
24	Healthcare decision	Decision-making on family’s healthcare (Respondent alone, Respondent & Husband/Partner, Respondent & another person, Husband/Partner alone, Someone else, Do not have husband)

Supplementary Table 2 Baseline Characteristics and Descriptive Statistics for Penta 3 Vaccination Dropout Prediction

**Features**		**Penta3 Vaccination Status**			
		**Completed**		**DroppedOut**	
		Frequency	Percentage	Frequency	Percentage
Mother’s age	15-19	3769	7.2	1037	11.3
	20-24	13763	26.2	2593	28.2
	25-29	13456	25.6	2223	24.1
	30-34	10802	20.6	1693	18.4
	35-39	7112	13.5	1132	12.3
	40-44	2968	5.7	420	4.6
	45-49	638	1.2	108	1.2
Place of residence	Urban	13017	24.8	2052	22.3
	Rural	39491	75.2	7154	77.7
Mother’s highest educational level	No education	9522	18.1	2332	25.3
	Primary	26011	49.5	4403	47.8
	Secondary	14041	26.7	2084	22.6
	Higher	2934	5.6	387	4.2
Country of residence	Tanzania	5008	9.5	684	7.4
	Burundi	6553	12.5	610	6.6
	Ethiopia	2678	5.1	1178	12.8
	Kenya	8553	16.3	1585	17.2
	Madagascar	4089	7.8	958	10.4
	Malawi	8054	15.3	1047	11.4
	Rwanda	4211	8.0	274	3.0
	Uganda	6150	11.7	1744	18.9
	Zambia	4618	8.8	715	7.8
	Zimbabwe	2594	4.9	411	4.5
Total number of under-five children	0	1130	2.2	268	2.9
	<5	51009	97.1	8859	96.2
	5-10	351	0.7	73	0.8
	>10	18	0.0	6	0.1
Household family size	<5	27710	52.8	4444	48.3
	6-10	22367	42.6	4203	45.7
	11-15	2108	4.0	485	5.3
	>16	323	0.6	74	0.8
Media exposure	No Exposure	18382	35.0	3809	41.4

	Have Exposure	34126	65.0	5397	58.6
Wealth Index	Rich	19543	37.2	2955	32.1
	Middle	10003	19.1	1606	17.4
	Poor	22962	43.7	4645	50.5
Mother’s cigarette smoking habit	Don't Smoke	52223	99.5	9143	99.3
	Smoke	285	0.5	63	0.7
Health care access	No problem	23083	44.0	3826	41.6
	Big problem	29425	56.0	5380	58.4
Mother’s occupational status	Did not work	16328	31.1	3555	38.6
	Professional/technical/managerial	3337	6.4	471	5.1
	Sales/Services	5740	10.9	1041	11.3
	Agriculture/Domestic	20418	38.9	3215	34.9
	Manual/Army	6685	12.7	924	10.0
Place of delivery	Home	8947	17.0	2665	28.9
	Gov't HF	41135	78.3	6131	66.6
	Private HF	1645	3.1	285	3.1
	Other	781	1.5	125	1.4
Had the baby a postnatal check within 2 months	No	37030	70.5	6645	72.2
	Yes	15478	29.5	2561	27.8
Place baby was first checked	Private Health Facility	120	0.2	17	0.2
	Home	2471	4.7	562	6.1
	Gov't Health Facility	11699	22.3	1800	19.6
	Other	57	0.1	10	0.1
	Did Not Have Check up	38161	72.7	6817	74.0
Time the mother received PN check-up immediately after delivery	Did not have PN CheckUp	37597	71.6	6779	73.6
	In less than 24 Hours	1399	2.7	319	3.5
	Within a week	6046	11.5	945	10.3
	In greater than one week	7466	14.2	1163	12.6
Who performed the postnatal checkup of the mother	Doctor	1909	3.6	310	3.4
	Midwife	9734	18.5	1497	16.3
	Nurse	1570	3.0	236	2.6
	Traditional Birth Attendant	938	1.8	292	3.2
	Community/Village Health Worker	384	0.7	64	0.7
	Other	143	0.3	24	0.3
	Did Not have Check up	37830	72.0	6783	73.7
Religion of the family	Christian	29202	55.6	4619	50.2
	Muslim	9685	18.4	1624	17.6
	Adventiste/Jehovah	8933	17.0	1900	20.6
	Traditional/Animist/No_ Religion	3957	7.5	913	9.9
	Other	731	1.4	150	1.6
Is the family covered by health insurance	No	46354	88.3	8682	94.3
	Yes	6154	11.7	524	5.7
	No	48030	91.5	8570	93.1

Was the baby born with a CS delivery	Yes	4478	8.5	636	6.9
Educational status of husband	No education	7415	14.1	1822	19.8
	Primary	20546	39.1	3433	37.3
	Secondary	12278	23.4	1935	21.0
	Higher	3885	7.4	543	5.9
	Do not have Husband	8384	16.0	1473	16.0
Occupational status of husband	Did not work	4168	7.9	916	10.0
	Professional/technical/managerial	4806	9.2	754	8.2
	Sales/Services	5318	10.1	1000	10.9
	Agriculture/Domestic	16817	32.0	2997	32.6
	Manual/Army	13048	24.8	2070	22.5
	Do not have Husband	8351	15.9	1469	16.0
Husband age	15-30	16333	31.1	3156	34.3
	31-45	23265	44.3	3760	40.8
	46-60	4082	7.8	719	7.8
	>61	506	1.0	108	1.2
	Do not have Husband	8322	15.8	1463	15.9
Earning of the mother	More than him	2138	4.1	316	3.4
	Less than him	13301	25.3	2059	22.4
	About the same	3601	6.9	577	6.3
	Do not have Husband/Living with partner	33468	63.7	6254	67.9
Who decides on the family's healthcare need	Respondent Alone	11175	21.3	1806	19.6
	Respondent & Husband/Partner	22816	43.5	3989	43.3
	Respondent and other person	0	0.0	0	0.0
	Husband/Partner alone	10033	19.1	1925	20.9
	Someone else	161	0.3	23	0.2
	Do not have Husband	8323	15.9	1463	15.9

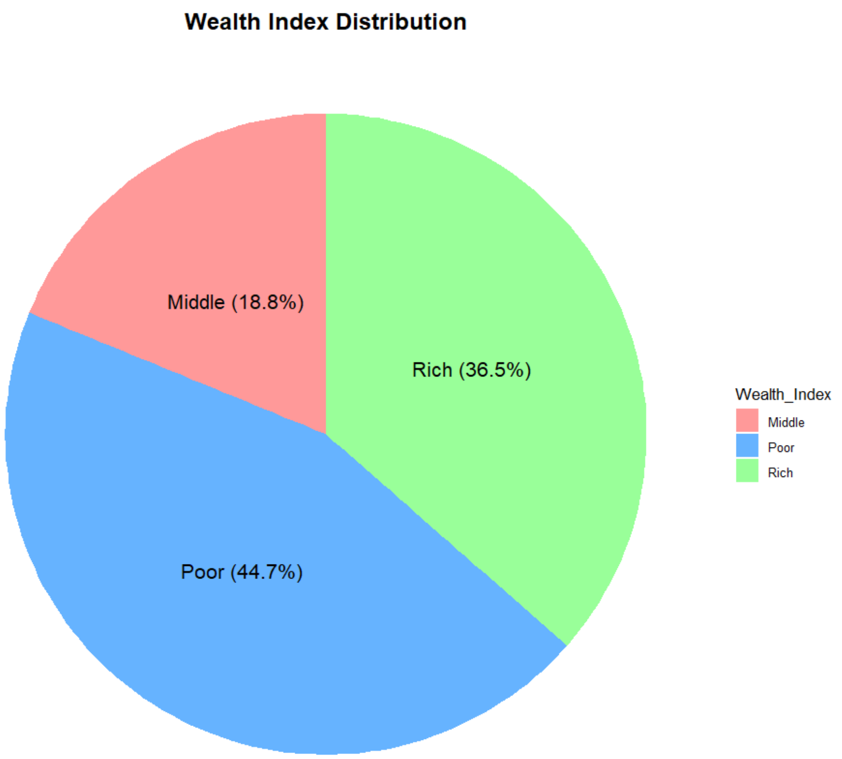

Supplementary Figure 1 The distribution of wealth index as a key feature

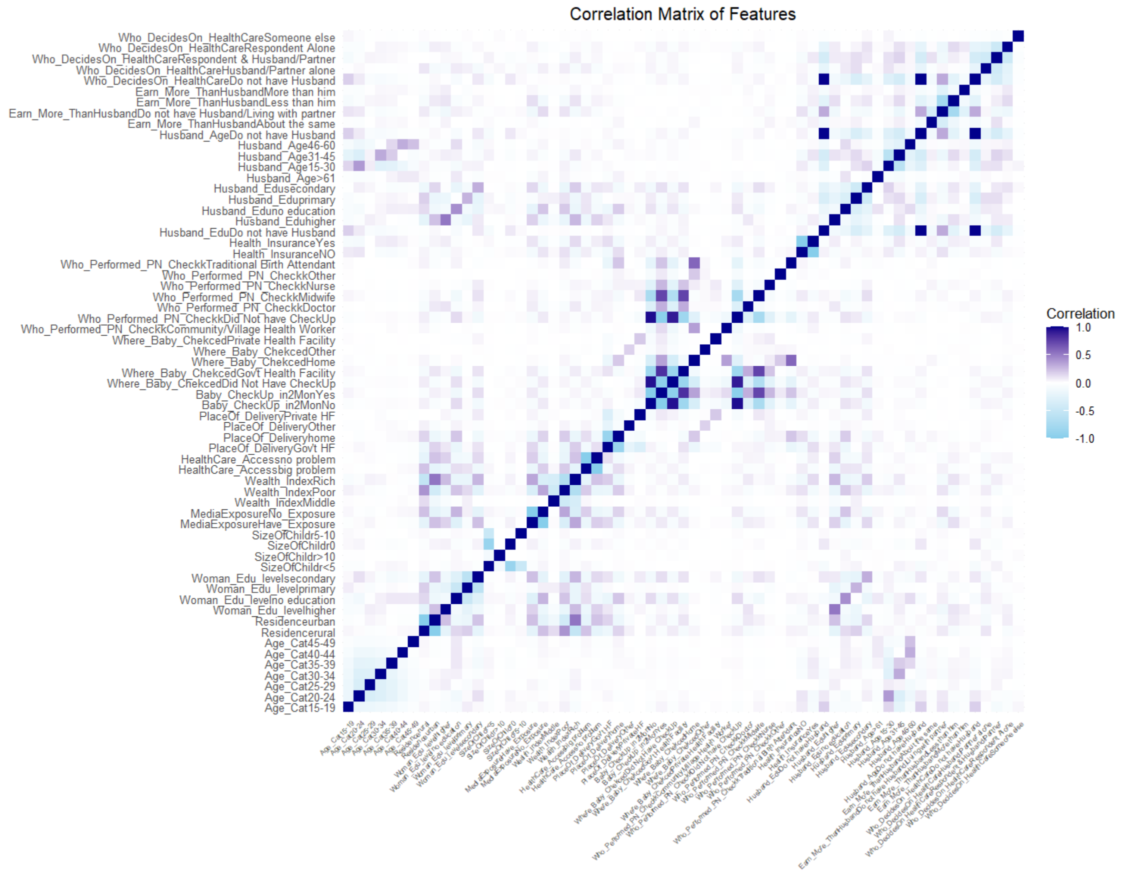

Supplementary Figure 2 Correlation Matrix of features to predict Penta3 Vaccination Dropout

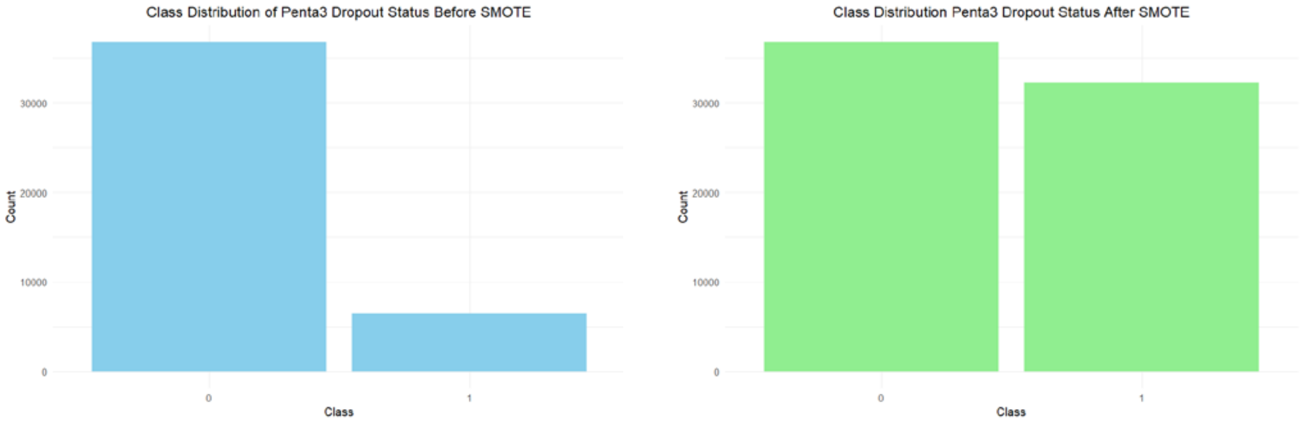

Supplementary Figure 3 Distribution of Penta 3 Vaccination Dropout Status Before and After Application of SMOTE
